# Adjusting for spatial variation when assessing individual-level risk: A case-study in the epidemiology of snake-bite in Sri Lanka

**DOI:** 10.1371/journal.pone.0223021

**Published:** 2019-10-03

**Authors:** Dileepa Senajith Ediriweera, Anuradhani Kasthuriratne, Arunasalam Pathmeswaran, Nipul Kithsiri Gunawardene, Shaluka Francis Jayamanne, Kris Murray, Takuya Iwamura, David Griffith Lalloo, Hithanadura Janaka de Silva, Peter John Diggle

**Affiliations:** 1 Centre for Health Informatics, Biostatistics and Epidemiology, Faculty of Medicine, University of Kelaniya, Ragama, Sri Lanka; 2 Department of Public Health, Faculty of Medicine, University of Kelaniya, Ragama, Sri Lanka; 3 Department of Parasitology, Faculty of Medicine, University of Kelaniya, Ragama, Sri Lanka; 4 Department of Medicine, Faculty of Medicine, University of Kelaniya, Ragama, Sri Lanka; 5 Department of Infectious Disease Epidemiology, School of Public Health, Faculty of Medicine, Imperial College London, St Mary’s Campus, London, United Kingdom; 6 Grantham Institute—Climate Change and the Environment, Imperial College London, South Kensington, London, United Kingdom; 7 Faculty of Life Sciences, School of Zoology, Tel Aviv University, Tel Aviv, Israel; 8 Liverpool School of Tropical Medicine, Liverpool, United Kingdom; 9 CHICAS, Lancaster University Medical School, Lancaster, United Kingdom; Johns Hopkins Bloomberg School of Public Health, UNITED STATES

## Abstract

**Background:**

Health outcomes and causality are usually assessed with individual level sociodemographic variables. Studies that consider only individual-level variables can suffer from residual confounding. This can result in individual variables that are unrelated to risk behaving as proxies for uncaptured information. There is a scarcity of literature on risk factors for snakebite. In this study, we evaluate the individual-level risk factors of snakebite in Sri Lanka and highlight the impact of spatial confounding on determining the individual-level risk effects.

**Methods:**

Data was obtained from the National Snakebite Survey of Sri Lanka. This was an Island-wide community-based survey. The survey sampled 165,665 individuals from all 25 districts of the country. We used generalized linear models to identify individual-level factors that contribute to an individual’s risk of experiencing a snakebite event. We fitted separate models to assess risk factors with and without considering spatial variation in snakebite incidence in the country.

**Results:**

Both spatially adjusted and non-adjusted models revealed that middle-aged people, males, field workers and individuals with low level of education have high risk of snakebites. The model without spatial adjustment showed an interaction between ethnicity and income levels. When the model included a spatial adjustment for the overall snakebite incidence, this interaction disappeared and income level appeared as an independent risk factor. Both models showed similar effect sizes for gender and age. HEmployment and education showed lower effect sizes in the spatially adjusted model.

**Conclusions:**

Both individual-level characteristics and local snakebite incidence are important to determine snakebite risk at a given location. Individual level variables could act as proxies for underling residual spatial variation when environmental information is not considered. This can lead to misinterpretation of risk factors and biased estimates of effect sizes. Both individual-level and environmental variables are important in assessing causality in epidemiological studies.

## Introduction

Both individual characteristics and environmental factors are important to individual health and disease outcomes [1] [2]. Environmental factors can be biological, chemical, physical and social, any of which can lead to disease [3]. Environmental factors typically show geographical or seasonal variation. Studies that consider only the individual-level variables when evaluating disease causation consequently suffer from residual confounding [1]. This can result in individual variables that are unrelated to risk behaving as proxies for uncaptured environmental information, leading to misinterpretation [4]. Environmental factors have also been shown to have interactions with individual-level variables [5], suggesting a need to consider both individual-level and environmental data in epidemiological studies aimed at understanding disease risk [1].

Assessing both individual and environmental factors help to avoid overestimation of individual risk-factor effects [6]. Environmental variables can only explain the average risk at a given location, whereas individual-level variables capture heterogeneity of risk at a location due to differences in behavior and individual characteristics. Estimates obtained without adjusting for spatial variation in risk could be confounded by location, hence masking individual-level causal effects. Estimating the effects of individual-level factors whilst adjusting for geographical variation in epidemiological studies is analogous to the long-established practice of blocking to control for extraneous variation in a randomized field experiment.

Sri Lanka reports 80 000 snakebites annually, with substantial geographical and seasonal variation in incidence [7] [8]. There are more than 100 snake species in the country, amongst which six are considered as medically important (*Naja naja*, *Bungarus ceylonicus*, *Bungarus caeruleus*, *Daboia russelii*, *Echis carinatus* and *Hypnale hypnale*). *Daboia russelii* is the largest venomous snake in Sri Lanka and is widely distributed in the country. *Naja naja* is the largest elapid in Sri Lanka and shows a wide distribution in the country. *Bungarus ceylonicus* and *Bungarus caeruleus* have the most potent venom and can be found in and around human habitats in the dry zone of the country. *Hypnale hypnale* is considered to be a moderately venomous snake and is responsible for 35% to 45% of all human bites. These snakes can commonly be found in human habitats including rubber, tea, coconut and cocoa plantations. *Echis carinatus* is only responsible for 1% to 2% of bites and is confined to the arid dry zones of the country. *Naja naja*, *Bungarus ceylonicus* and *Bungarus caeruleus* cause neurotoxic effects and *Daboia russelii*, *Echis carinatus* and *Hypnale hypnale* mainly show haematological abnormalities [7] [9] [10].

Previous local hospital data have shown that the majority of snakebite victims are middle-aged males [11] [12] [13]. Similar findings have been reported in other countries [14] [15] [16] [17]. There is a scarcity of epidemiological data on risk factors for snakebite, as most snakebite studies are still conducted in hospital settings [18]. In one study, Caiaffa et al investigated the risk factors for snakebite by using hospital patients who do not have a history of snakebites as controls. They reported that agricultural workers and people living in rural areas are high-risk groups for snakebite [19]. Health-seeking behaviour following snakebite shows considerable variation, and hospitals do not provide healthcare service to all snakebite victims [10]. Therefore, hospital data on snakebite are potentially biased, limiting their suitability for assessing risk factors.

There is a paucity of literature on individual-level risk factors for snakebite and this has been highlighted as an area that requires further research [20]. Previous epidemiological studies have presented the collective demographic characteristics of snakebite victims, but have not evaluated individual-level risk factors [7] [14] [15] [16] [17]. Although both geographical and seasonal variation of snakebites have been studied in Sri Lanka, individual-level risk factors have not been evaluated [7] [8]. In this study, we evaluate the individual-level risk factors of snakebite risk in a community-based national representative survey in Sri Lanka. We considered snakebite incidence at the sampled location as an environmental variable, to highlight the effect of spatial confounding on determining risk factors, and the importance of considering both individual and environmental variables in assessing causality in epidemiological studies.

## Materials and methods

### Epidemiological data

Epidemiological snakebite data were collected by conducting an island-wide community-based “National Snakebite Survey”. The survey was designed to sample 1% of the Sri Lankan population. This was a cross sectional study and multi-stage cluster sampling technique was used to sample population. A multi-stage cluster sampling technique was used to sample 0.8% of the Sri Lankan population. Sri Lanka has nine provinces, with between two and five districts per province. Each district is further subdivided into a national total of 14 022 Grama Niladari divisions. Grama Niladari divisions are the smallest administrative divisions in Sri Lanka. Each Grama Niladari division was considered as a potential cluster for the survey. In each province, 125 clusters were proportionally allocated among the districts according to their population sizes, and the allocated number of clusters randomly selected from the complete list of Grama Niladari divisions in each district. Within each cluster, 40 consecutive households from the electoral register were sampled, with the first household randomly selected. The survey included all the permanent members in the sampled households. The interviewer used a questionnaire to collect data from a responsible adult household member. All snakebite events that occurred during the preceding year were recorded, along with individual demographic data on the household members and the geo-locations of the household. The survey was conducted from August 2012 to June 2013. It covered all the nine provinces and all the 25 districts in the country. Details of the “National Snake Survey” has been published in our previous publication (i.e. Ediriweera, Dileepa Senajith, et al. "Mapping the risk of snakebite in Sri Lanka-a national survey with geospatial analysis." PLoS neglected tropical diseases 10.7 (2016): e0004813). A location map of Sri Lanka and the administrative divisions are shown in S1 and S2 Figs.

This study was approved by the Ethics Review Committee of the Faculty of Medicine, University of Kelaniya (Ref: P 06/01/2012). All interviews were conducted after obtaining informed written consent. Approval from District and Divisional level public administrators was obtained for conducting the community-based survey. No animals were used in the study.

### Snakebite incidence data

Sri Lanka does not possess cluster-level information on snakebite. Therefore, we extracted cluster-level snakebite incidence from our previously published snakebite incidence map of Sri Lanka [7]. These incidence estimates were obtained using a geostatistical model that considered population density, height above sea level, occupation distribution and climatic zone. Snakebite incidence at the centroid of each cluster was attached to each sampled individual, which implicitly assumes that the incidence does not vary within clusters. The median area of a cluster is approximately 2.0 (IQR: 0.9–4.2) km^2^. Details of the geostatistical model is given in S1 Appendix.

### Statistical methods

For exploratory analysis, generalized additive models were used to identify non-linear associations in explanatory variables. Generalized linear models were then used to model the probability of snakebite at individual level. All models were fitted using the R programming language version 3.4.2 [21]. We considered age, sex, ethnicity, religion, education, employment and income as exposure variables. We treated age as a continuous variable and the remainder as categorical variables. Categorical variables were collapsed when we found no differences between levels, testing at the conventional 5% level. Multicollinearity between exposure variables were assessed during the model building using variance inflation factor to avoid multicollinearity in the fitted models. The survey collected data from 165 665 individuals (about 0.8% of the Sri Lankan population). After removal of records with missing data, 158 066 records were available for the analysis.

Separate models were fitted to investigate individual-level risk factors with and without adjusting for spatial variation in snakebite incidence, so as to assess the impact of the spatial adjustment on the interpretation of individual-level risk-factors; we refer to these models as spatially adjusted and spatially non-adjusted models, respectively. Log likelihood ratio test and z statistic were considered to select variables in the spatially non-adjusted model and spatially adjusted models respectively.

The spatially adjusted model is a generalized linear mixed model that allows for extra-binomial and spatially correlated variation in risk. Snakebite incidences obtained from geostatistical models are associated with standard errors and conclusions drawn from a single sample of a predictive distribution of snakebite incidence could be error bound. Therefore, we used multiple imputation of these estimates when fitting the spatially adjusted model that included snakebite incidence as an exposure variable. This was done by adopting a two-stage algorithm. Stage 1 generates 10,000 samples from the predictive distribution of the overall risk-map derived from a geostatistical model [7] fitted using the R package PrevMap [22]. This gave 10,000 imputed snakebite incidences from the predictive distribution at each location. Stage 2 passes each sample to a generalized linear model to account for individual-level variation in risk within locations. Stage 2 assumes that the responses from different individuals are conditionally independent given the spatial risk surface (i.e. the covariates including the mapped risk surface are sufficient to explain the spatial variation in risk to an individual). Final estimates and standard errors of regression parameters for individual risk-factors were then calculated using a standard result in probability theory (formula 1 and 2):

E(β^)=E(E(β^|U)) (1)

Var(β^)=Var(E(β^|U))+ E(Var(β^|U)) (2)

where $\hat{\beta }$ is a parameter estimate, U an imputed value of a risk factor and a vertical bar denotes conditioning.

To apply this result, let U_i_ denote the i^th^ imputed map, ${\hat{\beta }}_{i}$ the corresponding regression parameter estimate and v_i_ the variance of ${\hat{\beta }}_{i}$ as reported by the generalized linear model software. Then, the final estimate, ${\hat{\beta }}_{i}$ is the sample mean of the 10,000 ${\hat{\beta }}_{i}$ and its variance is the sum of two components: the sample variance of the ${\hat{\beta }}_{i}$ and the sample mean of the v_i._

Estimated cluster level random effects of the spatially adjusted model were then used to assess for the presence of any residual, unexplained spatial correlation. This was done by calculating the empirical variograms of the predicted random effects and for 1000 random permutations of these. From these random permutations, we obtained pointwise 95% tolerance limits under the assumption of spatially uncorrelated random effects. The variogram of the random effects was not contained within the envelope of the tolerance limits indicating a possible spatial correlation (S3 Fig). We therefore estimated the covariance parameters of spatial correlation using PrevMap package [22]. The estimated variance of the Gaussian process is much smaller than the estimated variance of the nugget effect (0.003 and 0.226 respectively). Therefore, we concluded that the spatial component of the residual, unexplained cluster-level spatial variation is negligible (S1 Table) and there is no evidence of lack of fit on the spatially adjusted model [23].

## Results

### Demography

The survey sampled 165,665 individuals (0.8% of the population of Sri Lanka) living in 44136 households from 1118 clusters. The median and interquartile range of participants’ age were 35 and 20–52, respectively. 50.1% of participants were male. The majority of participants’ ethnicity was Sinhalese and the majority religion was Buddhism. Nearly 25.9% had been educated up to G.C.E. Advanced Level or above, and 68.4% were field workers including farmers. The survey reported 695 snakebite events during the year preceding interview. Demographic characteristics of survey participants are given in Table 1.

**Table 1:** Demographic characteristics of participants in the national snakebite study, Sri Lanka.

Characteristics	Number (%) or Median (IQR)
Age (years)	35 (20–52)
< 15	28 663 (17.3%)
15–24	25 502 (15.4%)
25–34	26 449 (16.0%)
35–44	25 090 (15.1%)
45–54	23 508 (14.2%)
55–64	18 884 (11.4%)
> 64	17 559 (10.6%)
Sex	
Males	82 888 (50.1%)
Females	82 705 (49.9%)
Ethnicity	
Sinhalese	123 839 (74.8%)
Tamils	29 852 (18.0%)
Muslims	11 841 (7.1%)
Other	97 (0.1%)
Religion	
Buddhist	120 644 (72.8%)
Catholic/Christian	6 527 (3.9%)
Hindu	26 444 (16.0%)
Islam	11 985 (7.2%)
Other	22 (0.1%)
Education	
No schooling	5658 (0.1%)
Primary	33 535 (20.2%)
Secondary	77 403 (46.8%)
Advanced level	42 888 (25.8%)
Above Advanced level	6060 (0.1%)
Employment	
Field workers	113 310 (68.4%)
Others	52 345 (31.6%)
Monthly income (Sri Lankan rupees)	
<5000	23 570 (14.5%)
5000–10 000	28 533 (17.5%)
10 000–20 000	50 451 (31.0%)
20 000–35 000	50 733 (31.2%)
>35 000	9446 (5.8%)
Estimated snakebite incidence (per 100,000) at sampled locations 336 (216–468)	

### Individual-level variable analysis from spatially non-adjusted model

In the generalized linear model without adjusting for the local snakebite incidence, age, sex, ethnicity, education, employment and income were associated with snakebite at individual level (Table 2). Age showed a non-linear association with snakebites, males showed higher risk compared to females (Odds ratio = 1.57, 95% CI: 1.34–1.83), and those who have studied below G.C.E. Advanced Level have higher risk than the rest (Odds ratio = 1.55, 95% CI: 1.24–1.90). Employment (Field workers vs non-field workers, Odds ratio = 1.58, 95% CI: 1.30–1.92) appeared as an independent risk factor and there was a strong interaction between ethnicity and income levels.

**Table 2:** Fitted model for predicting a snakebite event without considering snakebite incidence.

	Estimate	Std. Error	z value	*P*(>|z|)	Odds ratio (95% CI)
(Intercept)	-1.14e+01	4.30e-01	-2.64e+01	< 0.001	-
Age	2.38e-01	1.71e-02	1.39e+01	< 0.001	-
Age^2	-2.17e-03	1.75e-04	-1.23e+01	< 0.001	-
Sex (male)	4.50e-01	7.95e-02	5.66e-00	< 0.001	1.57 (1.34–1.83)
Ethnicity (Sinhalese)	-4.88e-01	1.98e-01	-2.47e-00	0.014	0.61 (0.42–0.90)
Education (advanced level or above)	-4.42e-01	1.01e-01	-4.35e-00	< 0.001	1.55 (1.24–1.90)
Employment (field workers)	4.56e-01	1.01e-01	4.54e-00	< 0.001	1.58 (1.30–1.92)
Income (5-20k)	-9.98e-01	1.99e-01	-5.02e-00	< 0.001	0.37 (0.25–0.54)
Income (>20k)	-1.33e+00	3.40e-01	-3.92e-00	< 0.001	0.26 (0.14–0.51)
Ethnicity (Sinhalese) : Income (5-20k)	1.14e+00	2.62e-01	4.36e-00	< 0.001	3.12 (1.87–5.22)
Ethnicity (Sinhalese) : Income (>20k)	1.74e+00	3.79e-01	4.59e-00	< 0.001	5.70 (2.71–11.96)

Variable units and categories: Age (years); Sex includes males and females; Employment includes field workers and others; Education includes below Advanced level and Advanced level or above; Income includes <5k, 5 – 20k and > 20k; Ethnicity includes Sinhalese and others

**Fig 1:**
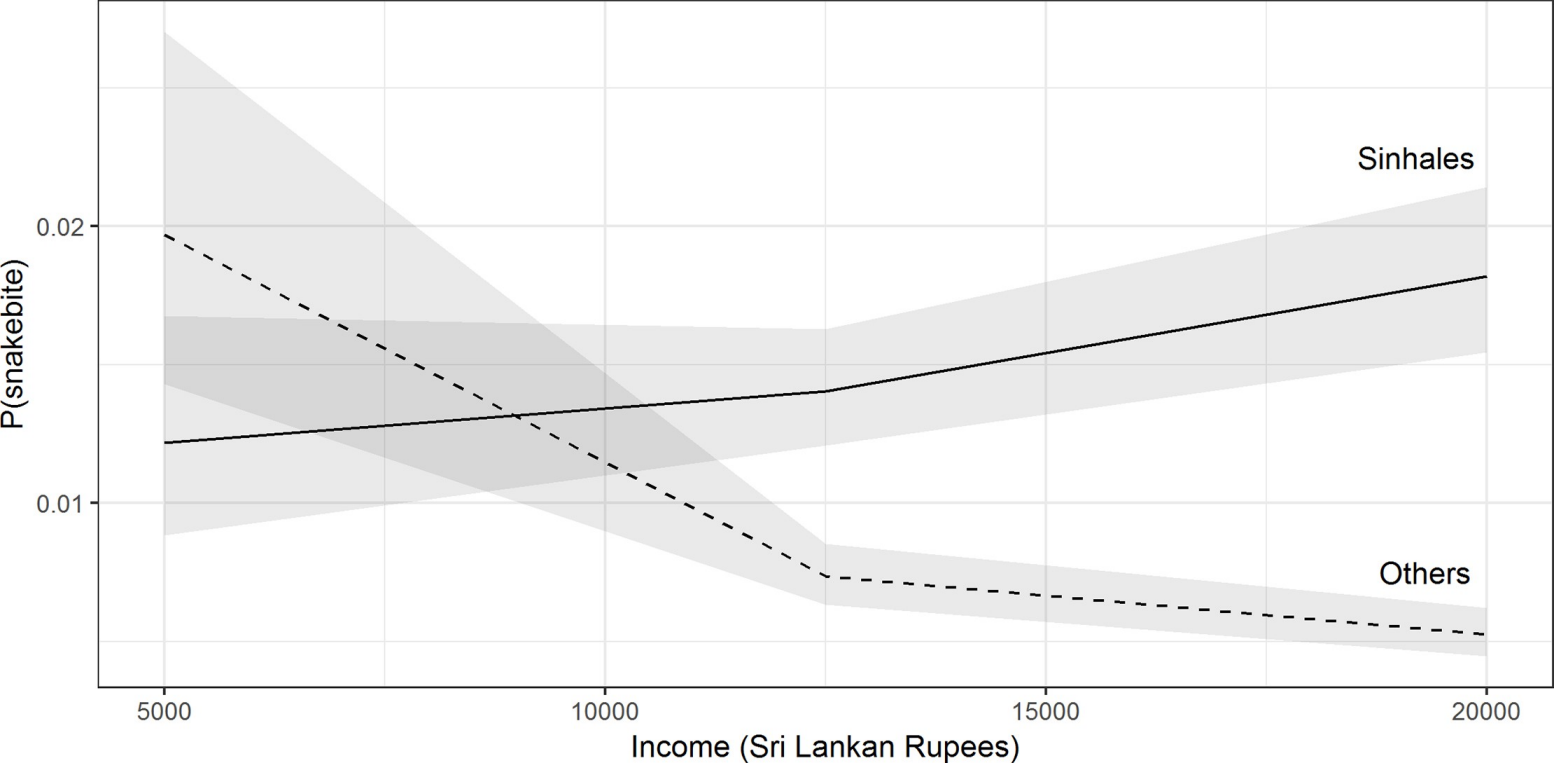
Interaction plot between ethnicity and income. Interaction plot between ethnicity and income for 50-year-old male farmers who had a low level of education. Predicted probability of snakebite among Sinhalese increased along with income rise (solid line) and the probability of snakebite decreased in Non-Sinhalese along higher income categories (dashed line). Grey colour bands represent the 95% confidence intervals.

For Sinhalese, snakebite risk increased along with income, whereas in other ethnic groups snakebite decreased as income increased (Fig 1). According to the fitted model, maximum snakebite risk was observed among the low-educated, field-working, Sinhalese, males aged 55 years, for whom the fitted probability of being bitten by a snake in a year was 0.012 (95% CI: 0.009–0.017).

### Individual-level variable analysis from spatially adjusted model

Individual risk factor analysis after adjusting for spatial variation of snakebite incidence (i.e. spatially adjusted model) showed that snakebite risk is associated with an individual’s age, sex, employment, education level, income level and local snakebite incidence (Table 3).

**Table 3:** Fitted model for predicting a snakebite event after adjusting for snakebite incidence.

	Estimate	Std. Error	z value	*P*(>|z|)	*Odds ratio* *(95% CI)*
(Intercept)	-1.24e+01	4.38e-01	-2.82e+01	< 0.001	-
Age	2.39e-01	1.72e-02	1.38e+01	< 0.001	-
Age^2	-2.16e-03	1.76e-04	-1.23e+01	< 0.001	-
Sex (male)	4.49e-01	8.01e-02	5.60e+00	< 0.001	1.57 (1.34–1.83)
Employment (field workers)	3.56e-01	1.03e-01	3.45e+00	< 0.001	1.43 (1.17–1.75)
Education (advanced level or above)	-2.87e-01	1.04e-01	-2.76e+00	0.003	1.33 (1.09–1.63)
Income (5–20k)	-4.68e-01	1.16e-01	-4.04e+00	< 0.001	1.59 (1.30–2.00)
Income (>20k)	-2.57e-01	1.24e-01	-2.07e+00	0.019	1.29 (1.01–1.65)
Incidence	2.48e-03	2.52e-04	9.86e+00	< 0.001	1.0024 (1.0019–1.00029)

Variable units and categories: Age (years); Sex includes males and females; Employment includes field workers and others; Education includes below Advanced level and Advanced level or above; Income includes <5k, 5 – 20k and > 20k; Incidence (estimated number of bites per 100,000 people in a given location)

**Fig 2:**
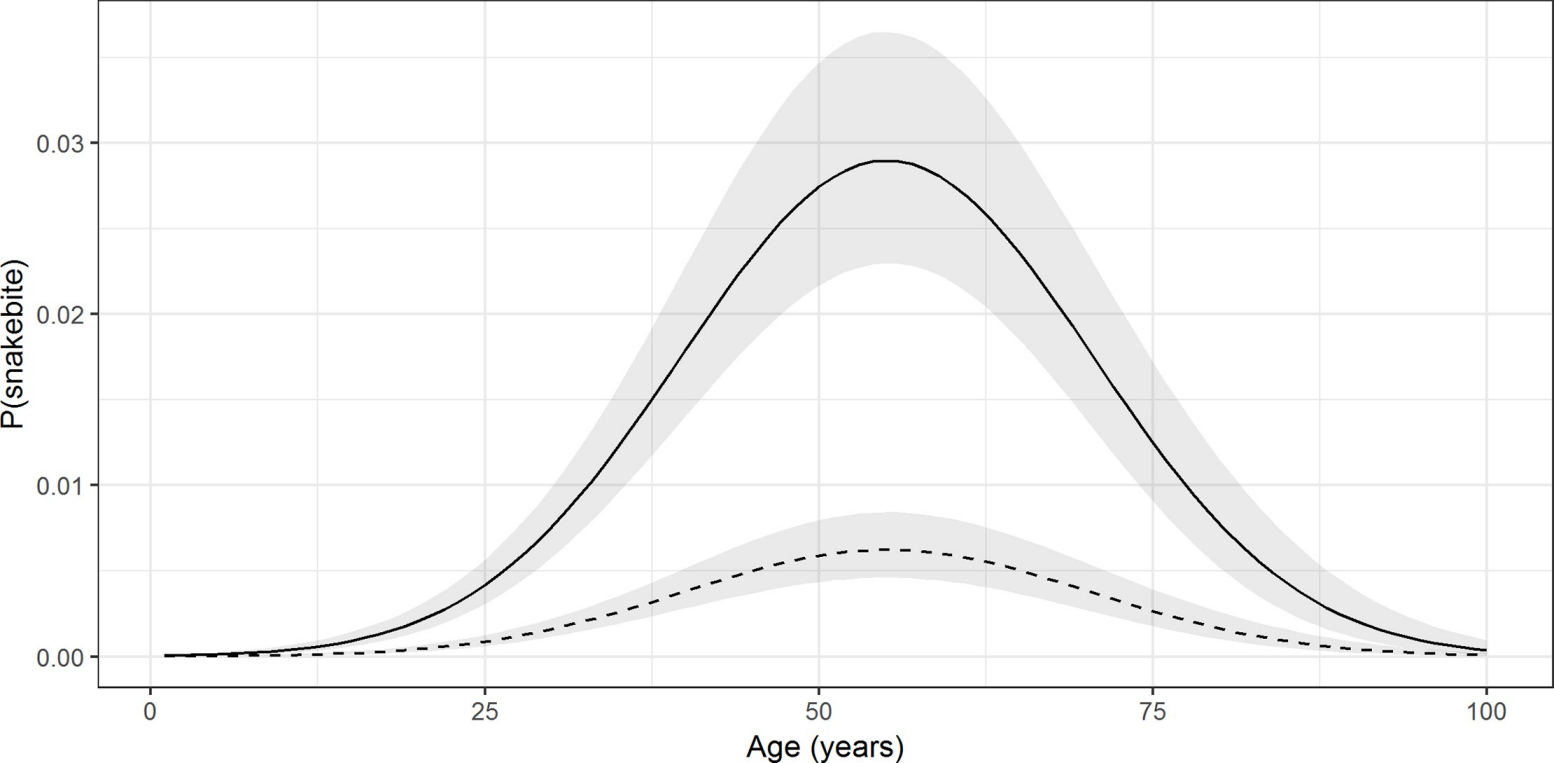
Probability of observing a snakebite along with age. Variation of probability of observing a snakebite along with age for a) low-educated, field-working, low-income males living in a high snakebite endemic area (solid line). b) higher educated, non-field working, middle-income females living in the same area and (dashed line). Grey colour bands represent the 95% confidence intervals.

Snakebites showed a non-linear association with age. Snakebite risk was higher in middle-aged people compared both to younger and to older (Fig 2). Males showed higher snakebite risk compared to females (Odds ratio = 1.57, 95% CI: 1.34–1.83) and field workers showed higher risk compared to non-field workers (Odds ratio = 1.43, 95% CI: 1.17–1.75). Individuals who had not studied up to G.C.E. Advanced Level showed a higher risk for snakebite than those who had studied up to or beyond G.C.E. Advanced Level (Odds ratio = 1.33, 95% CI: 1.09–1.63). The lowest income group (i.e. less than Rs. 5000 per month) showed the highest snakebite risk compared to the middle-income group (i.e. between Rs. 5000 to 20 000; Odds ratio = 1.59, 95% CI: 1.30–2.00) and to the high-income group (i.e. more than Rs. 20,000 per month; Odds ratio = 1.29, 95% CI: 1.01–1.65). According to the spatially adjusted model, in high endemic snakebite areas (e.g. Northcentral province with 600 bites per 100 000), maximum snakebite risk was noted among the low-educated, field working, low-income 55-year-old males, for whom our estimated probability of being bitten was 0.029 (95% CI: 0.023–0.036). In the same areas, educated, non-field working, middle income 55-year-old females showed 0.006 (95% CI: 0.005–0.008) probability of being bitten by snakes.

### Difference between spatially non-adjusted and adjusted models

**Fig 3:**
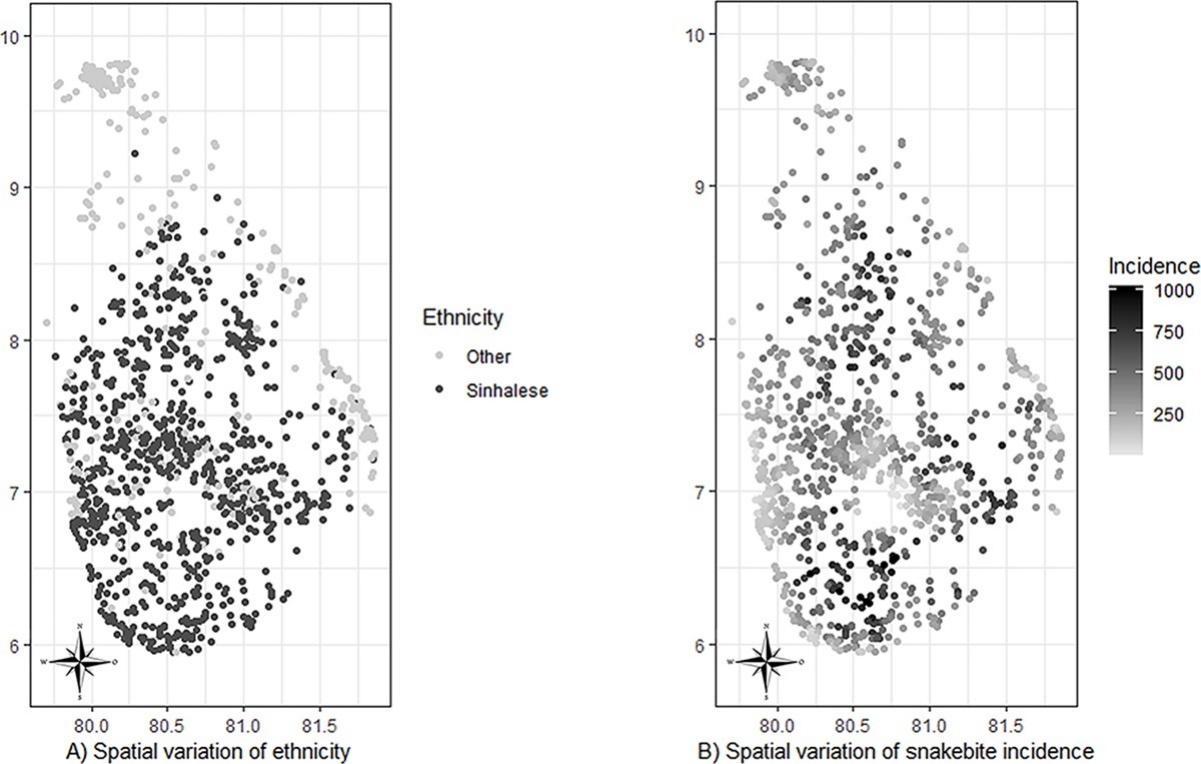
Spatial variation of ethnicity and snakebite incidence. Spatial variation of A) ethnic groups and B) snakebite incidence in the sampled locations. Sinhalese lived in areas with relatively high snakebite incidence compared to other ethnic groups.

Both models showed age, sex, employment and education to be risk factors for snakebite. Ethnicity was a risk factor in the spatially non-adjusted model and showed a significant interaction with income categories. Ethnicity did not appear as a risk factor in the spatially adjusted model whereas income appeared an independent risk factor. Both ethnicity and snakebite incidence demonstrated spatial variation across the country (Fig 3). Sinhalese lived in relatively high snakebite incidence areas compared to other ethnic groups (median (IQR): 372 (244–508) per 100,000 among Sinhalese versus 250 (184–328) per 100,000 among other ethnic groups, Therefore, it is likely that the ethnicity has acted as a proxy variable to underlying spatially varying snakebite incidence when the snakebite incidence is not considered (i.e. spatially non-adjusted model).

## Discussion

Both the individual-level characteristics and local snakebite incidence showed important effects on determining snakebite risk. Individual-level covariates remain significant after adjusting for local snakebite incidence. This highlights that the local snakebite risk (i.e. geographical location) does not full account for variation in individual level risk. Local snakebite incidence provides only an average snakebite risk on a given location and individual level characteristics, such as individual behavior, cause heterogeneity in risk at a location. Location of an individual could be either an inherent risk factor itself or a proxy for unmeasured, spatially structured risk factors, in which case the spatial adjustment helps to estimate the effect size of the individual level covariates that are confounded by their locations [24]. This ambiguity is unavoidable in a non-randomised observational study. Hence, whilst we consider it important to consider the effects of both individual-level and geographical risk factors on health outcomes their interpretation needs to be informed by context. Here, there is strong face validity to the proposition that snakebite risk depends both on environmental factors, not all measured, that vary between locations (here, clusters), and on individual factors that vary within locations.

Both spatially adjusted and non-adjusted models showed that middle-aged, males, field workers and low-educated individuals have high risk for snakebites. Low-income level appeared as an independent risk factor for snakebite in the spatially adjusted model. In the non-spatially adjusted model, all the exposure variables except religion showed association with snakebites, and the same model showed an interaction between ethnicity and income levels. Religion is closely related to ethnicity in Sri Lanka and the non-significance of religion can be explained by the presence of ethnicity in the non-spatially adjusted model. It is likely that the significance of all other variables, including the interaction term, is at least partially attributable to the attempt to capture the residual variability in the data rather than reflecting direct causation. The interaction between ethnicity with income was non-significant in the spatially adjusted model. It is likely that in the model without spatial adjustment ethnicity acts as proxy variable for geographical variation in snakebite incidence, as ethnicity shows a noticeable geographic variation over the country [25]. A previous national mortality study in India has reported an association between snakebite deaths and religion [26]. It is possible that this result also reflects an underlying geographical variation in snakebite risk rather than a causal effect. Therefore, failure to consider both ecological and individual characteristics can lead to misinterpretation of risk factors that are acting as proxies for unmeasured information [4].

Both spatially adjusted and non-adjusted models give similar parameter estimates and standard errors for gender and for the quadratic effect of age. Neither gender nor age is likely to be spatially confounded in the current setting. In contrast, parameter estimates for employment and education were higher in the spatially non-adjusted model compared to the spatially adjusted model. This could reveal either an overestimation of risk in the spatially non-adjusted model [6] or dilution of risk estimates in the spatially adjusted model [24]. On the other hand, the spatially non-adjusted model estimated a lower overall probability for snakebites for the high-risk individuals compared to the spatially adjusted model (i.e. 0.029 (95%CI: 0.023–0.036) vs 0.012 (95% CI: 0.009–0.017) respectively).

Individual level covariates that are identified by this study (i.e. in the spatially adjusted model) are compatible with previous literature. Both local [11] [12] [13] and regional studies [14] [15] [16] [17] have shown that the males and middle-aged have high snakebite risk. The active workforce of Sri Lanka comprises 63.5% of males and the majority of the workforce belong to middle-aged groups [27], therefore males and middle-aged individuals are likely to encounter more exposures to snakebites while working in rural industries. Previous studies have also reported a high percentage of farmers [12] [28] [29] and low educational levels among snakebite victims who are admitted to hospitals [28]. Snakebite is a disease of poverty; low income and low socio-economic individuals have been identified as risk factors for snakebite [30].

The limitations of our study include the following. Firstly, our analysis relies on recall, rather than direct observation, of snakebite events. Secondly, the snake bitten pattern varies between species, but we do not have data on the biting species. Thirdly, the national snakebite survey captured only the employment status of individuals, not the victim’s activity at the time of the bite. Fourthly, we did not consider the seasonal variation of snakebite into the analysis and estimates can be subjected to seasonal bias. Finally, the true snakebite incidence at each sampled location is unknown, hence we used multiple imputation to estimate parameters in the spatially adjusted model in order to allow for the uncertainty in the estimated incidence map.

In conclusion, we highlight the importance of considering environmental information in additional to individual-level factors when designing epidemiological studies so as to avoids the latter acting as proxies for the former, leading in turn to biased estimates of effect sizes. We suggest that it is important to consider both spatial and non-spatial information in future health research [31]. Our results show that males, middle-aged individuals, field workers, individuals with low-education and income are at high risk of snakebite. This highlights the importance of conducting educational programs and mass media campaigns, to educate the public on vulnerable groups for snakebite and to promote safety measure to avoid snakebites.

## Supporting information

S1 Fig Location map of Sri Lanka. (TIFF)

S2 Fig Administrative boundaries and sampled Grama Niladari Divisions. a) Administrative boundaries of Sri Lanka. Dark lines demarcate the provinces and grey lines demarcate the districts of Sri Lanka. b) Locations of the Grama Niladari Divisions sampled by the National Snakebite Survey of Sri Lanka.
(TIFF)

S3 Fig Empirical variograms of the estimated cluster level random effects of the spatially adjusted model. The black line indicates the empirical variogram of the predicted random effects. Dashed lines indicate the 95% pointwise tolerance envelope for the empirical variograms of 1000 random permutations of the random effects.
(TIFF)

S1 Table Estimated covariance parameters of spatial correlation. (DOCX)

S1 Appendix Geostatistical modelling of snakebite incidence. (DOCX)

S1 Data Individual level data. (CSV)

S2 Data Data for snakebite incidence estimation. (CSV)
