# Supplementary figures and images for: Adjusting for spatial variation when assessing individual-level risk: A case-study in the epidemiology of snake-bite in Sri Lanka
Source: PLoS One. 2019 Oct 3;14(10):e0223021. doi: 10.1371/journal.pone.0223021 (PMC6776347; doi:10.1371/journal.pone.0223021)

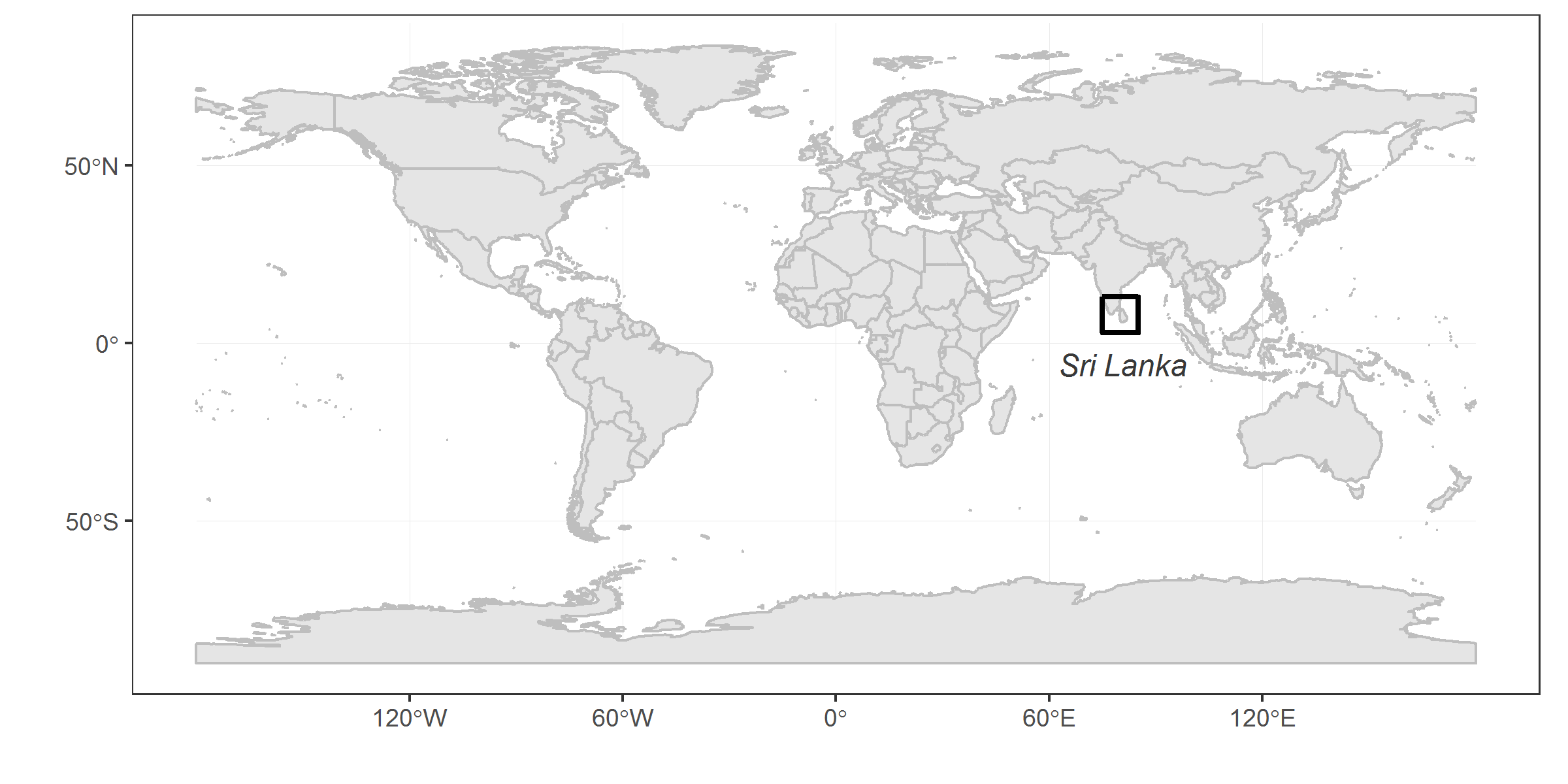

Supplement: S1 Fig — (TIFF) [file pone.0223021.s001.tiff]

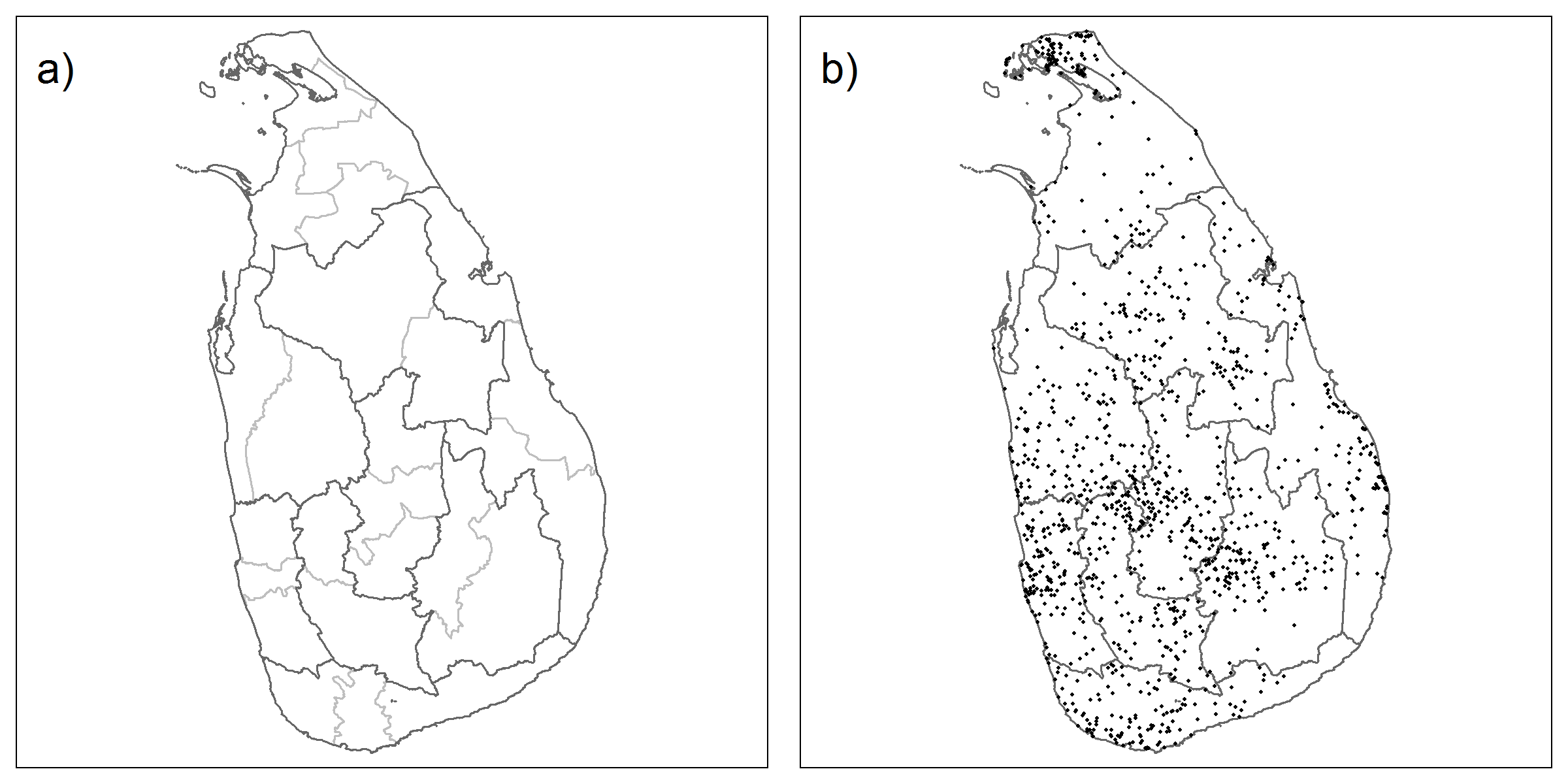

Supplement: S2 Fig — a) Administrative boundaries of Sri Lanka. Dark lines demarcate the provinces and grey lines demarcate the districts of Sri Lanka. b) Locations of the Grama Niladari Divisions sampled by the National Snakebite Survey of Sri Lanka. (TIFF) [file pone.0223021.s002.tiff]

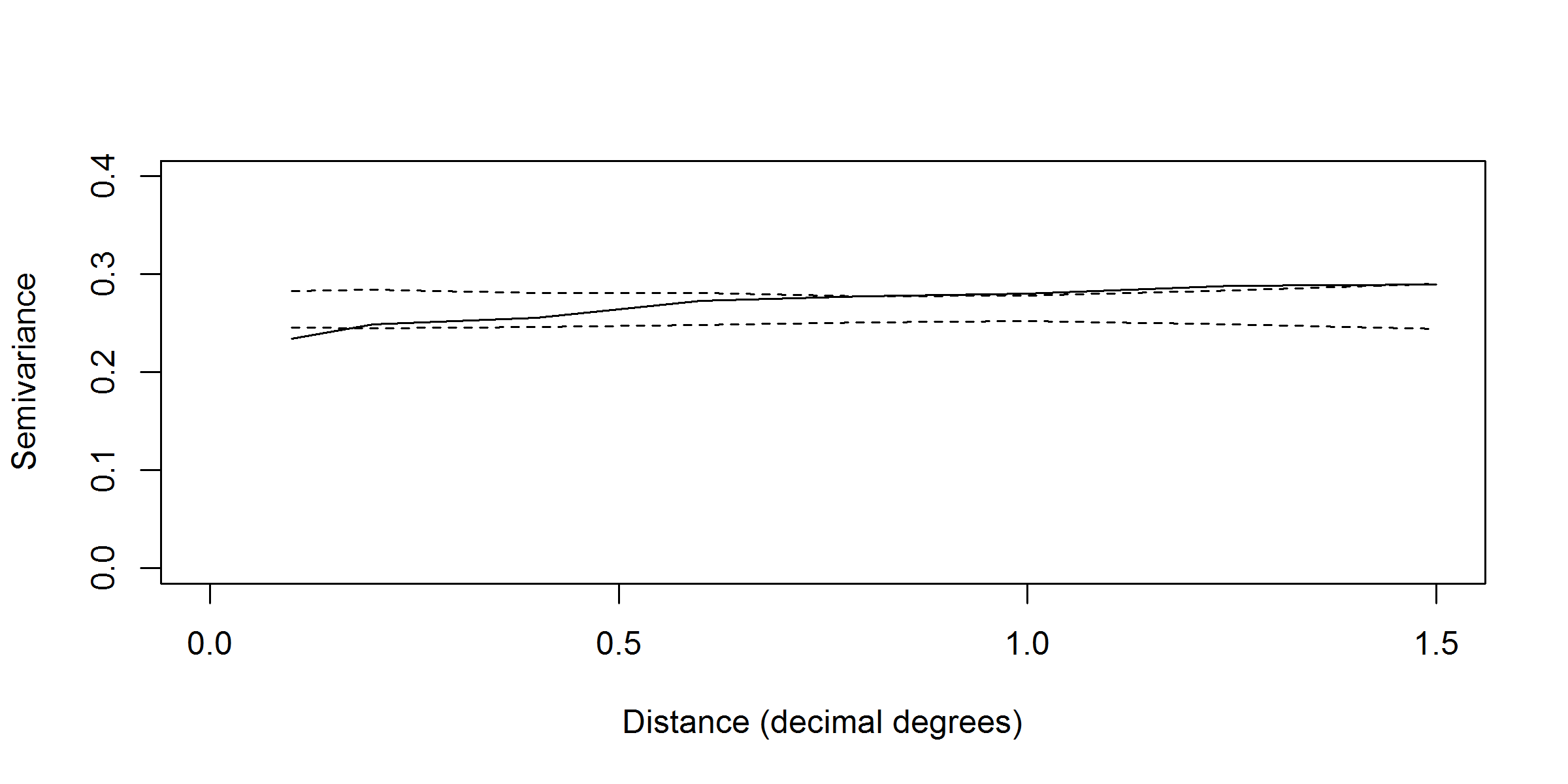

Supplement: S3 Fig — The black line indicates the empirical variogram of the predicted random effects. Dashed lines indicate the 95% pointwise tolerance envelope for the empirical variograms of 1000 random permutations of the random effects. (TIFF) [file pone.0223021.s003.tiff]
