# Supplementary material for: Adjusting for spatial variation when assessing individual-level risk: A case-study in the epidemiology of snake-bite in Sri Lanka
Source: PLoS One. 2019 Oct 3;14(10):e0223021. doi: 10.1371/journal.pone.0223021 (PMC6776347; doi:10.1371/journal.pone.0223021)
Supplement: S1 Table — (DOCX) [file pone.0223021.s004.docx]

**Table 1. Estimated covariance parameters of spatial correlation**

| Parameter | Estimate | Std. Err. |
| --- | --- | --- |
| σ^2^  (variance of the Gaussian process) | 0.003 | 280.877 |
| φ (scale of the spatial correlation) | 0.228 | 5.120 |
| τ^2^  (variance of the nugget effect) | 0.266 | 284.197 |
