# Supplementary material for: Adjusting for spatial variation when assessing individual-level risk: A case-study in the epidemiology of snake-bite in Sri Lanka
Source: PLoS One. 2019 Oct 3;14(10):e0223021. doi: 10.1371/journal.pone.0223021 (PMC6776347; doi:10.1371/journal.pone.0223021)
Supplement: S1 Appendix — (DOCX) [file pone.0223021.s005.docx]

# S1 File: Geostatistical modelling of snakebite incidence

Snakebite incidence estimates were obtained using a standard geostatistical model (equation 1)

Log {p(x)/ [1- p(x)]} = α + f(explanatory variables) + S(x) equation (1)

At location x_i_, y_i_ is the number of snakebites out of n_i_ individuals. The y_i_ are independent binomial events conditional on an unobserved spatial stochastic process S(x), hence the conditional mean number of snakebite incidence at location x_i_ depends on explanatory variables observed at location x_i_ and on S(x_i_), and p(x) is the probability that a person at location x will have a snakebite.

Explanatory variables for the geostatistical model were identified by conducting an explanatory analysis with generalized linear models and generalized additive models. Piece-wise functions were adopted when non-linear associations were identified. Population density, height above sea level, occupation distribution and climatic zone were considered as explanatory variables.

The role of the term S(x) is to identify spatial variation in risk that is not explained by measured explanatory variables. S(x) is modelled as a Gaussian process with mean zero, variance σ^2^ and correlation structure corr[S(x), S(xˊ)]= exp (-u/φ), where u is the distance between x and xˊ and φ is the scale of spatial correlation. The term S(x) in captures the residual spatial variation after adjusting for the covariates.

Initial values for regression coefficients and covariance parameters were obtained from the respective generalized linear model fit with piece-wise models and least-squares estimation of the empirical variogram respectively.

A Markov chain Monte Carlo (MCMC) algorithm was used to simulate the samples required for Monte Carlo maximum likelihood estimation. Autocorrelograms and trace plots were assessed to determine the required number of iterations. Series of simulated samples were produced as follows: the numbers of iterations for simulated samples were predefined as 1100, 5000, 10 000, 25 000, 50 000 and 75 000. The first 10% of the samples were discarded as burn-in to minimize dependence on the initial values. Subsequent samples were thinned so as to obtain 1000 samples from the total simulated samples. Thinning reduces the storage requirement and the dependence among sampled values. After accessing autocorrelograms and trace plots (Figure 1), 50 000 simulated samples, with 5000 burn in and retention of every 45th sample were selected for parameter estimation and spatial prediction. .

| 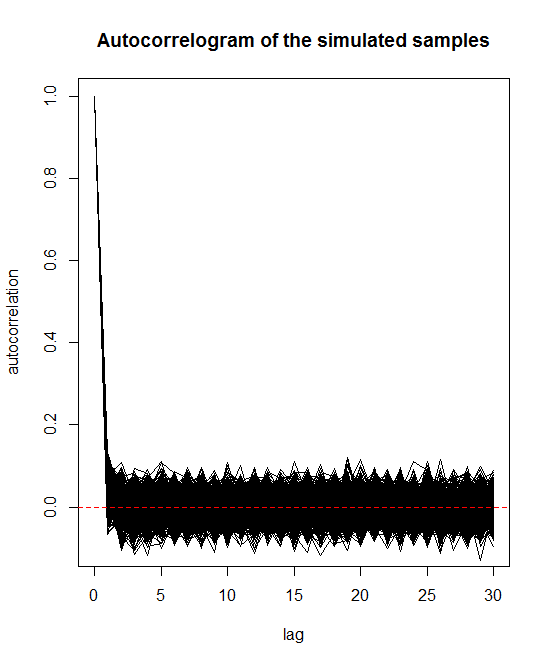 | 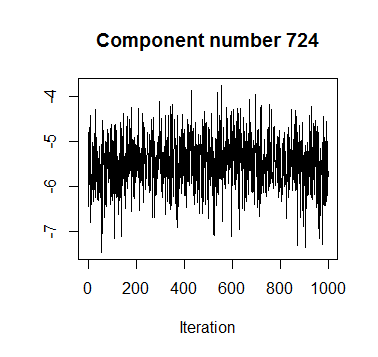 |
| --- | --- |
| (A) Autocorrelogram of the simulated samples for snakebite incidence | (B) Trace plot of the simulated samples for snakebite incidence |

**Fig 1: Convergence diagnostics:** (A) autocorrelogram and (B) trace plot of simulated samples for snakebite incidence.

The fitted geostatistical model for snakebite incidence is summarised in table 1.

**Table 1 Parameter estimates from geostatistical model for snakebite incidence**

| Variable | Estimate | Std. Error | Z value | P value |
| --- | --- | --- | --- | --- |
| (Intercept) | -6.6239 | 0.13456 | -49.2246 | <0.001 |
| Elevation | 0.0032 | 0.00079 | 4.0771 | <0.001 |
| Elevation more than 160 meters | -0.0046 | 0.00085 | -5.4389 | <0.001 |
| Climate zone 2 (Intermediate) | 0.2225 | 0.10564 | 2.1068 | 0.035 |
| Climate zone 3 (Wet) | 0.5586 | 0.11763 | 4.7492 | <0.001 |
| Population density | -0.0002 | 0.00002 | -10.8101 | <0.001 |
| Percentage of agricultural workers | 7.2235 | 1.33660 | 5.4043 | <0.001 |
| Percentage of agricultural workers > 9% | -6.4166 | 1.40170 | -4.5778 | <0.001 |
|  |  |  |  |  |
| Covariance parameters Matern function (kappa=0.5) | | | | |
| sigma^2^ | 0.189 | 0.8778 |  |  |
| Phi | 0.091 | 2.5249 |  |  |
| tau^2^ | 0.304 | 1.5944 |  |  |

**Reference:**

- Ediriweera, Dileepa Senajith, et al. "Mapping the risk of snakebite in Sri Lanka-a national survey with geospatial analysis." PLoS neglected tropical diseases 10.7 (2016): e0004813
